# In‐vivo plasma biomarker concentrations correlate with postmortem AD‐neuropathology in the oldest old

**DOI:** 10.1002/alz.090703

**Published:** 2025-01-09

**Authors:** Linda M.C. Lorenz, Susan K. Rohde, Maruelle C. Luimes, Patricia Fierro‐Hernández, Annemieke J.M. Rozemuller, Netherlands Brain Bank, Jeroen J.M. Hoozemans, Charlotte Teunissen, Henne Holstege

**Affiliations:** ^1^ Alzheimer Center Amsterdam, Department of Neurology, Amsterdam Neuroscience, Vrije Universiteit Amsterdam, Amsterdam UMC, Amsterdam, Netherlands, Amsterdam Netherlands; ^2^ Amsterdam Neuroscience, Neurodegeneration, Amsterdam, Netherlands, Amsterdam Netherlands; ^3^ Section Genomics of Neurodegenerative Diseases and Aging, Department of Clinical Genetics, Vrije Universiteit Amsterdam, Amsterdam UMC, Amsterdam, Netherlands, Amsterdam Netherlands; ^4^ Department of Pathology, Amsterdam Neuroscience, Amsterdam UMC, Amsterdam, Netherlands, Amsterdam, Netherlands, Amsterdam Netherlands; ^5^ Amsterdam UMC, location VUmc, Amsterdam, Netherlands, Amsterdam, Netherlands, Amsterdam Netherlands; ^6^ Netherlands Institute for Neuroscience, Amsterdam Netherlands; ^7^ Neurochemistry Laboratory, Department of Clinical Chemistry, Amsterdam UMC, Vrije Universiteit Amsterdam, Amsterdam Neuroscience, Amsterdam Netherlands; ^8^ Delft Bioinformatics Lab, Delft University of Technology, Delft, The Netherlands, Delft, Netherlands, Delft Netherlands

## Abstract

**Background:**

There has been ongoing uncertainty to what extent plasma biomarkers are representative of neuropathological markers of AD pathology in the oldest old. A complicating factor is that with increasing age, Aβ‐ and Tau pathologies accumulate in the brains of cognitively healthy individuals to highly variable levels (Zhang & Ganz et al., 2022). The unique availability of both antemortem plasma biomarker concentrations and postmortem neuropathology in self‐reported cognitively healthy oldest old, offers a chance to explore whether levels of plasma biomarkers can serve as in‐vivo proxy of AD neuropathology.

**Methods:**

We evaluated postmortem neuropathology of 48 centenarian brains, with a median MMSE score of 26 (range:16–30) at blood draw. We quantified plasma biomarkers Aβ_40_, Aβ_42_, Aβ_42/40_, pTau181, NfL and GFAP on the Simoa platform. The median interval between blood‐sample collection and brain donation was 1.3 (range:0–5.1) years. In addition to conventional spatio‐temporal staging systems such as Thal‐Aβ phase (median 2, range:0‐5) and Braak‐NFT stage (median 3, range:2‐5), we also quantified pathological load by measuring the percentage area of immunopositivity for total Aβ (range:0–9.0%), Aβ_40_ (range:0–3.3%), Aβ_42_ (range:0–11.5%), pTau‐217 (range:0–1.8%) and AT8 (pTau‐202/205; range:0–49.2%) in the cortex of the temporal pole. Partial Spearman correlations were conducted adjusted for age, sex, and time interval.

**Results:**

Plasma pTau‐181 correlated with Aβ pathology loads, namely Aβ_40_ (r=0.48, p<0.001), Aβ_42_ (r=0.41, p=0.005) and total Aβ (r=0.48, p<0.001), as well as with spatio‐temporal Thal‐Aβ phase (r=0.45, p=0.003). Plasma pTau‐181 correlated with quantitative pTau pathology loads, specifically pTau‐217 (r=0.48, p=0.001) and AT8 (r=0.41, p=0.005), but not with spatio‐temporal Braak‐NFT stage (r=0.17, p=0.290). Furthermore, both plasma NfL and plasma GFAP also correlated with pTau‐217 load in the brain (r=0.30, p=0.0496; r=0.30, p=0.044, respectively). Plasma Aβ_40_, Aβ_42_, Aβ_42/40_ did not correlate with any neuropathology measure.

**Conclusions:**

Results reveals plasma pTau‐181, but not plasma Aβ, as a reliable indicator for both Aβ and pTau pathology loads in the temporal pole of the oldest old. The 100‐plus study cohort provides a promising opportunity to study the correlation between different pTau epitopes and the presence of pTau levels, as well as the presence of Tau pathology, including NFTs, neuropil threads, pre‐tangles and ARTAG.

